# Mechanisms of Resistance to Conventional Therapies for Osteosarcoma

**DOI:** 10.3390/cancers13040683

**Published:** 2021-02-08

**Authors:** Louise Marchandet, Morgane Lallier, Céline Charrier, Marc Baud’huin, Benjamin Ory, François Lamoureux

**Affiliations:** 1 UMR1238, Phy-OS, Sarcomes Osseux et Remodelage des Tissus Calcifiés, INSERM, Université de Nantes, 44035 Nantes, France; louise.marchandet@univ-nantes.fr (L.M.); morgane.lallier@univ-nantes.fr (M.L.); celine.charrier@univ-nantes.fr (C.C.); marc.baudhuin@univ-nantes.fr (M.B.); Benjamin.Ory@univ-nantes.fr (B.O.); 2 CHU de Nantes, 44035 Nantes, France

**Keywords:** Osteosarcoma, chemotherapy resistance, chemotherapy circumvent

## Abstract

**Simple Summary:**

Osteosarcoma (OS), the most common primary bone tumor, mainly affects children and adolescents. Unfortunately, in some cases, the absence of response to chemotherapy agents is observed, leading to metastases development and death of the patient. Resistance is one of the biological processes at the origin of therapeutic failure. In order to improve the therapeutic management of patients, it is necessary to identify and better understand the mechanisms underlying resistance. Here, we summarize molecular mechanisms of OS resistance to conventional chemotherapy and list some strategies that overcome resistance.

**Abstract:**

Osteosarcoma (OS) is the most common primary bone tumor, mainly occurring in children and adolescents. Current standard therapy includes tumor resection associated with multidrug chemotherapy. However, patient survival has not evolved for the past decades. Since the 1970s, the 5-year survival rate is around 75% for patients with localized OS but dramatically drops to 20% for bad responders to chemotherapy or patients with metastases. Resistance is one of the biological processes at the origin of therapeutic failure. Therefore, it is necessary to better understand and decipher molecular mechanisms of resistance to conventional chemotherapy in order to develop new strategies and to adapt treatments for patients, thus improving the survival rate. This review will describe most of the molecular mechanisms involved in OS chemoresistance, such as a decrease in intracellular accumulation of drugs, inactivation of drugs, improved DNA repair, modulations of signaling pathways, resistance linked to autophagy, disruption in genes expression linked to the cell cycle, or even implication of the micro-environment. We will also give an overview of potential therapeutic strategies to circumvent resistance development.

## 1. Introduction

Osteosarcoma (OS) is the most common primary bone tumor representing approximately 30% of bone sarcomas, and mainly affecting children and adolescents with an 18-years incidence peak [1]. The OS worldwide incidence rate is estimated to 4 cases per million per year [2]. Genetic factors may increase the risk of OS. A small percentage of patients with genetic changes or mutations are at higher risk for OS. Rare hereditary conditions due to specific genetic mutations, such as Li-Fraumeni syndrome, can also increase the risk of OS [3,4]. OS is characterized by formation of immature bone or osteoid tissue by tumor cells associated with areas of peri-tumor osteolysis. In 80% of patients, preferred anatomical sites of tumor development are metaphysis of long bones and, mainly, in areas of rapid bone growth. Indeed, 40% of OS are located at the femur, 20% at the tibia, and finally 10% at the humerus [5,6]. This tumor can also occur in the axial skeleton and soft tissue in 20% of cases. It is well described that OS originates from mesenchymal stem cells (MSCs) or osteoblasts and can be divided into different subtypes that are osteoblastic, chondroblastic, and fibroblastic [7,8,9]. At the time of diagnosis, there is a 5-year survival rate of around 75% for localized forms of OS (80% of patients). However, for patients with metastases, mainly pulmonary, on diagnosis (20% of patients), the 5-year survival rates dramatically decrease to 20%.

Until the 1970s, the only therapeutic management of OS was surgical and sometimes radiotherapy. It is important to note that OS are quite resistant to radiotherapy [10,11]. Surgery alone, which consisted of amputating or removing the tumor, did not reduce mortality below 80% [12]. Indeed, tumor excision only leads to a survival rate of around 20% at 5 years [13]. Since then, the use of chemotherapy agents has increased the survival rate of patients with OS and reduced amputations, and thus improved limb salvage. Indeed, the long-term survival rate is now 75% for patients with non-metastatic disease compared to 20% before the 1970s [14,15]. However, the long-term survival rate is still low for patients with metastatic or recurrent disease. Furthermore, nearly 85% of patients undergoing resection since the year 2000 have been able to keep their limbs [16]. The first chemotherapy protocols were established by Dr. Rosen and included high-doses of methotrexate, cyclophosphamide, bleomycin, and vincristine preoperatively and post-surgical chemotherapy with doxorubicin [17]. Treatments consisted of neo-adjuvant chemotherapy following by surgical resection and adjuvant chemotherapy. The objectives of neoadjuvant chemotherapy are firstly to damage tumor cells at the primary site in order to reduce tumor size before surgery. It also allows the eradication of micrometastases but also the assessment of the histological response of the tumor to chemotherapy. This response is evaluated according to the necrosis rate present within the tumor, used as a prognostic factor. If the tumor has a necrosis rate greater than or equal to 90%, the patient is a good responder. However, if this rate is less than 90%, the patient is a bad responder [18]. Adjuvant chemotherapy may be adapted according to the observed necrosis rate. Most of the used molecules in chemotherapy protocols are a combination of cisplatin (CDP), doxorubicin (DOX), methotrexate (MTX), and ifosfamide (IFO).

Chemotherapy agents have different mechanisms of action. The combination of their modes of action, therefore, makes it possible to target tumor cells at several levels. MTX inhibits the proliferation of rapidly dividing cells by inhibiting the reduction of folic acid. DOX is a cytotoxic antibiotic, which is able to inhibit topoisomerase II, interpose with DNA and RNA polymerases, induce the formation of free radicals, and to bind to membranes. CDP is a cytostatic antineoplastic capable of binding and inhibiting DNA synthesis, which prevents DNA replication and, therefore, cell division. IFO is metabolized to mafosfamide, binding to DNA to block its replication, thereby preventing cell division [19,20,21]. However, current treatments have limits including insufficient efficacy for high-risk patients and low survival rates for patients with metastases at diagnosis. Moreover, some patients developed chemotherapy resistance, explaining in some cases relapses and tumor progression. Indeed, around 25% of patients classified as good responders to chemotherapy will still relapse. In addition, poor responders can quickly develop metastases leading to death. Two types of resistance should be distinguished: Intrinsic resistance and acquired resistance [22]. Intrinsic resistance is already pre-existing since resistant cells are already present within the tumor before any treatment [23]. At the time of chemotherapy administration, sensitive cells will be eliminated due to the toxic effects of drugs. However, tumor resistant cells exhibiting pre-existing genetic mutations or activation of different signaling pathways would be able to proliferate despite the presence of chemotherapy agents. Conversely, acquired resistance is induced by drugs and is emerged after therapies. During treatment, there is a gradual reduction of anticancer efficacy of drugs. Indeed, tumor cells will develop mechanisms such as activation of proto-oncogene, mutations, or altered expression levels of transport proteins or drug targets, and changes in the tumor microenvironment (TME) [24]. These adaptations subsequently allow tumor cells to resist chemotherapy. Resistance, therefore, results from genomic instability where resistant clones will be selected after chemotherapy treatment. Both types of resistance are the result of specific molecules or molecular signaling pathways present in OS cells [25]. Resistance is one of the biological processes at the origin of therapeutic failure. To overcome it, more effective treatments are urgently needed. Therefore, it is necessary to better understand and decipher molecular mechanisms of resistance to conventional chemotherapy in order to elaborate new strategies and to adapt treatments for patients, thus improving the survival rate. Although the disease-free survival rate has dramatically improved with the advent of chemotherapy, only 50% to 60% of tumors are chemosensitive [26].

This review will describe most of the molecular mechanisms involved in OS chemoresistance and will give an overview of potential therapeutic strategies to circumvent resistance development and to sensitize tumor cells to conventional chemotherapies. The phenomenon of chemoresistance can be due to a decrease in intracellular accumulation of drugs, inactivation of drugs, improved DNA repair, modulations of signaling pathways, resistance linked to autophagy, turbulence in gene expression linked to the cell cycle, or even an implication of the micro-environment [2,27].

## 2. Decrease the Intracellular Accumulation of Drugs

**Figure 1:**
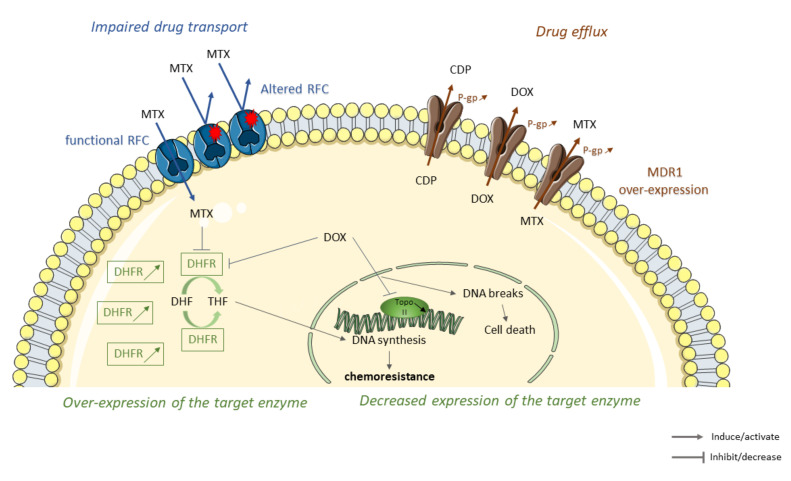
Some molecular mechanisms leading to the decrease of intracellular drug accumulation in osteosarcoma (OS) resistant tumor cells to conventional therapies. Sequence alterations of reduced folate carriers (RFC) lead to an impaired methotrexate (MTX) transport through the membrane cell, thereby reducing the accumulation of MTX inside the cell (in blue). Overexpression of members of ATP-binding cassette (ABC) family such as multidrug resistance protein 1 (MDR1) increases cisplatin (CDP,) doxorubicin (DOX), and methotrexate (MTX) efflux and elimination leading to chemoresistance (in brown). Overexpression or decrease expression of MTX/DOX-target enzymes lead to OS resistance. Chemotherapeutic agents, such as MTX or DOX, interact and inhibit target enzymes such as dihydrofolate reductase (DHFR). DHFR inhibition limits conversion of dihydrofolate (DHF) to tetrahydrofolate (THF), leading to a decrease in DNA synthesis and cell growth. DHFR overexpression in OS-resistant cells decreases MTX and DOX cytotoxicity leading to the emergence of resistance. Topoisomerase II (TopoII), an enzyme responsible for the topological state of DNA and, therefore, cell viability. DOX stabilize topoisomeraseII/DNA complex leading to cell death. Decrease expression of topoisomerase II decreases cytotoxicity of DOX and promotes the emergence of resistance (in green).

One of the first mechanisms used by tumor cells to deal with cytotoxic effects of chemotherapy agents is to decrease drug accumulation. For that, tumor cells can (1) establish inefficient transport by reducing the number of carriers on their surface [28], (2) initiate drug elimination by increasing drug efflux [29], (3) induce alterations in structure or expression of target enzyme [30] (Figure 1).

### 2.1. Impaired Drug Transport

One of the described resistance mechanisms to chemotherapy in OS is impaired drug transport, in particular, due to a decrease in transporters present on the surface of tumor cells. MTX is an antifolate drug entering into cells via three different ways: Folate receptors, proton-coupled folate transporter, and reduced folate carrier (RFC). MTX accumulates in cells and is then polyglutamylated. This form is preferably retained in cells. Then, MTX interacts and inhibits the dihydrofolate reductase (DHFR), an enzyme that converts dihydrofolate (DHF) to tetrahydrofolate (THF), which is a one-carbon donor for de novo purine and thymidine biosynthesis [31]. DHFR, therefore, plays a key role in intracellular folate metabolism and is essential for the synthesis of de novo DNA. Thus, the interaction between MTX and DHFR will prevent DNA synthesis and cell growth [32,33,34,35].

Some studies described RFC involvement in resistance to chemotherapy agents in OS. MTX intracellular concentration is lower in MTX resistant OS cells than in sensitive cells. Moreover, resistant cells present a lower RFC expression compared to sensitive cells [36]. The decrease in RFC expression in the tumor is associated with resistance to MTX in OS [37]. Samples with poor histological responses to preoperative chemotherapy, including high doses of MTX, show a higher decrease in RFC expression. A study showed a correlation between RFC protein level and histological response to chemotherapy [38]. In fact, tumors with a poor response to MTX have a significantly lower level of RFC expression at the time of diagnosis compared to good responders who have a higher-level expression of this protein. However, the protein level of RFC was significantly higher in recurrent tumor specimens than primary OS biopsy samples at diagnosis. This means that after chemotherapy, tumor progression is associated with higher expression of RFC protein, which should make cells more sensitive to MTX [39]. Some sequence alterations have been shown to change the functionality of RFC protein. As an example, Ser46Asn, Ser4Pro, or Gly259Trp alterations confer a certain resistance to MTX. Indeed, these altered RFC proteins have a reduced MTX transport rate. MTX enters into cells with Ser46Asn, Ser4Pro, or Gly259Trp alterations but in a lower amount than sensitive cells, which results in only partial resistance. Samples presenting genetic alterations are also those which have a higher frequency of poor histological response to chemotherapy meaning that alterations in RFC gene could be a prognostic factor. Alterations in RFC could contribute to the lack of responses to MTX, and to varying degrees depending on alterations [40]. Analysis of RFC gene copy number did not show any difference between parental cell lines and MTX resistant cells, meaning that reduction in expression of the gene encoding RFC is not due to gene deletion [41]. Reduction in RFC expression may also be due to promoter methylation and polymorphism in 3′UTR of RFC. Indeed, RFC expression is weaker in samples with more than 10% of methylation. In addition, samples with heterozygous polymorphism of 2582 T/G or 2617 C/T in the 3′UTR show a decrease in RFC expression [42].

In order to overcome resistance, an MTX-like drug, which does not require RFC for transport, has been developed. Trimetrexate was tested in a phase II clinical study including patients with relapsed or refractory OS. Five out of 38 patients (13%) treated with trimetrexate had a good response [43]. A combination of trimetrexate with high dose MTX is being tested in a phase I clinical study including patients with recurrent OS [44]. Other drugs with a higher affinity for RFC, such as antifolate pralatrexate, have been developed to compensate low expression of these carriers [45]. Pralatrexate has been evaluated in clinical trials and has been approved for the treatment of patients with refractory or recurrent peripheral T cell lymphoma [46]. Studies in patients with the bone disease may, therefore, be of interest.

Drug formulation techniques improve drug delivery efficacy. Liposomal encapsulation of DOX has been shown to be an effective mode of delivery. Its activity in relapsed sarcomas suggests that this technique may be able to overcome resistance [47]. An aerosolized formulation of CDP is investigated in a phase Ib/IIa clinical study, including OS patients with relapses limited to the lungs (sustain release lipid inhalation targeting [SLIT™] Cisplatin; Transave, Inc., Monmouth Junction, NJ, USA) [48].

A decrease in RFC expression reduces MTX transport within cells [36]. However, these changes ultimately confer a fairly low level of MTX resistance and cannot be responsible for the full observed resistance in vivo. In other words, in vivo intrinsic resistance is a combination of different molecular mechanisms among which this inefficient transport could be one of the contributing factors.

### 2.2. Drug Elimination

Increased drug efflux has been reported as partly responsible for OS resistance [49]. This phenomenon has been described for CDP, MTX, and DOX. Cancer cells exposed to one chemotherapy agent can develop resistance to many other anticancer drugs, called multidrug resistance (MDR) [50]. This acquired resistance is mainly due to overexpression of members ATP-binding cassette (ABC) family of efflux transporters [51]. There are efflux pumps such as multidrug resistance-associated proteins (MRP), noted ABCC1 and breast cancer resistance proteins, renamed ABCG2 and mitoxantrone resistance proteins (BCRP, MXR) [52]. The most typical transporter is the multidrug resistance protein 1 (MDR1), also named Glycoprotein (G-gp) [53,54]. These proteins are encoded by ABC subfamily B member 1 (ABCB1) [55]. Under physiological conditions, ABC transporters regulate cellular levels of hormones, lipids, ions, xenobiotics, and other molecules but also play a role in intracellular regulation of organelles such as mitochondrion, lysosome, endoplasmic reticulum, or Golgi apparatus [55]. The presence of these transporters on cancer cells membrane allows efflux of anticancer drugs outside of cells and thus leads to resistance [56].

OS cell lines exhibit a high level of expression of ABC transporters such as MDR1 [39]. Expression of 170 kDa plasma membrane glycoprotein of hamster, mouse, and human is increased in tumor cell lines. This protein has the same molecular size and some immunogenic homology as MDR1 and also confers MDR [57]. In human OS cells resistant to DOX, the association between MDR1 overexpression and reduced DOX accumulation within cells has been described [58]. MDR1 expression level is correlated with kinetic parameters of radiotracers mimicking DOX, meaning that the DOX chemosensitivity of OS cells depends on MDR1 expression [59]. Furthermore, MDR1 mRNA expression level increases gradually according to DOX concentration [60].

Indeed, some studies have found a correlation between high expression of ABC transporters and poor response to chemotherapy, poor outcome, and poor survival rate of patients [61]. Tumor expressing P-gp seems to be associated with an increase of metastases development and death risk [62]. Conversely, patients with better relapse-free, improved survival rate, and better long term outcomes do not overexpress P-gp [63,64]. In patients treated only with DOX and surgery, ABC transporters expression in tumor cells is significantly associated with a higher incidence of relapse and a worse outcome [65]. Serra et al. have shown that an increase of P-gp expression consorted with a tumor volume greater than 150 mL and a patient aged over 12 years at the time of diagnosis is associated with a high risk of recurrence [66]. It has also been found that polymorphisms in ABC members correlate with response to chemotherapy. OS treatment efficacy can be affected by certain polymorphism of ABC transporter genes, such as RS4148416 for ABCC3 and RS4148737, RS1128503, and RS10276036 for ABCB1. Clinical outcomes in the treatment of OS could be predicted by these different genetic polymorphisms [67].

Moreover, the implication of p53 in the correlation between ABC expression and poor outcomes have been shown. A study realized on 52 tumor specimens has shown that co-expression of P53 and P-gp may be an indicator of a short survival [68]. Conversely, a non-association between P-gp expression, p53 status, and the clinical outcome has been shown, but only an increase of P-gp in the tumor is significantly associated with a poor outcome [69]. However, Sorensen et al. showed that survival or treatment response cannot be predicted by P-gp expression and p53 status [70]. Furthermore, some studies have failed to couple P-gp expression with histological response, survival outcome, or event-free survival [71,72,73,74,75].

P-gp has been shown to be associated with a cytoskeleton linker named ezrin. This complex is located in plasma membrane lipid rafts. MDR observed in OS cell lines could also be due to this cytoskeleton linker. Indeed, ezrin inhibition increases drug sensitivity of tumor cells [76], and ezrin expression is associated with poor outcomes in OS patients [77].

ABC transporters can be targeted in order to overcome resistance. OS cell lines resistant to paclitaxel have a higher P-gp level and have also shown cross-resistance to other P-gp substrates such as DOX, docetaxel, and vincristine. However, in the presence of an inhibitor of P-gp, NSC23925, cells become sensitive to paclitaxel [78]. Tetrandrine (CBT-1), an alkaloid anti-inflammatory compound, prevents paclitaxel-induced MDR in OS cells [79]. P-gp may be upregulated by the growth factor pleiotrophin promoting DOX resistance. Chemosensitivity in OS cell lines was enhanced by pleiotrophin knockdown [80]. In two MDR OS cell lines (KHOSR2 and U-2OSR2), ABCB1 knock-out restored sensitivity to DOX but not to CDP [81]. Targeting ABCB1/ABCC1 by tetrandrine also restored DOX sensitivity in resistant OS cell lines, which could be an interesting alternative in treating refractory or recurrent OS [82]. In addition, MDR1 transcriptional activation may be inhibited by trabectedin, an alkaloid antineoplastic agent [83], which could be of interest in the treatment of resistant OS. Thus, targeting and altering transporters expression in combination with conventional treatments may be a potential therapeutic option to treat resistant OS.

### 2.3. Alterations in the Structure or Expression of the Target Enzyme

The resistance of some chemotherapeutic agents can also be explained by an increased level of target enzymes or a decrease of drug affinity due to mutations in these enzymes [25]. These variations have been observed in resistance to MTX and DOX. MTX resistant OS cell lines exhibit high expression of DHFR. Indeed, a relationship between high expression of DHFR in xenografts and emergence of resistance has been shown [84,85]. High expression of DHFR is rare in initial biopsy samples (10% of samples) but is very frequently observed in metastases (62% of samples), suggesting that this resistance mechanism appears during treatment. A correlation between retinoblastoma protein (pRb), playing a key role in the cell cycle, and the expression level but also enzymatic activity of DHFR has been shown. Indeed, cells with defective pRb have a higher DHFR mRNA level as well as a higher enzymatic activity than those with a functional protein. Cells with absent or abnormal pRB, therefore, tend to be resistant to MTX [86]. E2F transcription factors are reported to control Rb gene expression. In OS patients samples, a relationship has been shown between E2F transcription factors mRNA expression and DHFR mRNA expression [87]. In OS, gene duplication is not responsible for overexpression of the DHFR enzyme. In contrast, dysfunctional DHFR may be due to retinoblastoma (Rb) signaling aberrations. However, Serra and colleagues showed that the MTX-resistant OS cell line (U2-OS) overexpresses the DHFR gene due to an amplification of it [37,88]. Additionally, this increased level of DHFR expression may be linked to cells presenting an intact Rb gene pathway. Indeed, DHFR expression is negatively regulated by the Rb gene via E2F. Therefore, Rb can influence cell sensitivity to MTX targeting DHFR [41]. MiR-215 reduces DHFR expression but does not reduce resistance to MTX but conversely increases it. This can be explained because miR-215 overexpression led to a p53-dependent growth inhibition, and low-proliferating tumors are more resistant to MTX, an S-phase specific cytotoxic drug [89]. Thus, DHFR alterations would be associated with p53 status in OS and targeting DHFR may be a way to overcome the chemoresistance of OS.

DNA topoisomerases are nuclear enzymes, which regulate DNA topology. Topoisomerase II, the primary target of effective antitumor drugs such as DOX, alters the topological state of DNA. This mechanism is essential for cell replication and viability. A covalent complex between topoisomerase II and DNA is a necessary intermediate in DNA topoisomerization catalysis. Stabilization of this complex by DOX interferes with vital functions, such as DNA replication leading to cell death. There are two isoforms of topoisomerase II: Topoisomerase IIα and topoisomerase IIβ [90,91]. OS cell lines (143B, MG63, and HOS) showed a reduction of topoisomerase II expression in DOX-resistant cells compared to sensitive cells [85,92,93], suggesting that a decrease of DOX target is one of the mechanisms of drug resistance.

## 3. Drug Detoxification/Inactivation

Certain drugs, such as DOX or CDP, induce the formation and accumulation of reactive oxygen species (ROS) [94]. ROS can also be produced by normal cellular metabolism, generating energy in the biological system. Low levels of ROS are relatively well tolerated by cells, while a high concentration of ROS induces oxidative stress leading to cellular damage [95]. To limit this, cells have protective and detoxification mechanisms, including antioxidants such as gluthathione (GSH) and glutahtione-S- transferase (GST). Once DOX or CDP enters cells, they are activated through a series of spontaneous aquation reactions. Activated CDP and DOX are able to interact with DNA and produce DNA damage [96,97]. Drugs can then be inactivated by detoxification enzymes: GSH and GSTs. The main member of the GST family is GSTP1-1 enzyme [98,99].

High levels of GSTs are shown in a large number of cancers such as ovarian, colon, breast cancers [100]. Moreover, the role of GSTP1 in resistance to chemotherapy agents, in particular, CDP and DOX, has been shown in many studies [101] in various cancers such as ovarian cancer [102], colon cancer [103], breast cancer [104], lung cancer [105], and in OS [106,107]. OS cells treated with DOX or CDP exhibit an up-regulation of GSTP1 expression [106,107]. Moreover, GSTP1 expression is inducible by chemotherapeutic agents leading to resistance of tumor cells to chemotherapeutic agents. Conversely, more apoptosis and DNA damage were observed by suppressing GSTP1 expression [108]. Indeed, cell sensitivity to CDP increased following depletion of GSTP1 activity and, the degree of resistance seems to be proportional to the level of intrinsic glutathione content [109]. Patients with high levels of GSTP1 expression present a high relapse rate, suggesting that overexpression of this enzyme could be linked to a poor prognosis [107].

Studies carried out on samples from dogs suffering from spontaneous OS have also highlighted a correlation between the increase in GST activity in resistant cells and shorter median-remission and survival times [110], as well as a relationship between high levels of GSTP1 expression and remission or survival rate [111]. In human OS xenografts, higher levels of GST transcripts are correlated with cell resistance to chemotherapy [112]. At the time of the biopsy, before preoperative chemotherapy, 60 samples of human OS did not overexpress GST. However, at the time of surgery, GST was overexpressed, suggesting that overexpression of GST was associated with failure of preoperative chemotherapy [113].

Some genetic polymorphisms may have a role in response to chemotherapy and in prognosis in OS patients. The relationship between genetic polymorphisms of patients who received conventional chemotherapy treatment and histological response as well as the survival rate was described. Indeed, a poor histological response was increased in patients with GSTP1 c.313A > G p.I105V variants [114]. Patients with the GSTP1 Val/Val genotype would seem to have a lower survival rate compared with patients exhibiting the Ile/Ile genotype [115]. However, Yang et al. found that individuals with GSTP1 Val/Val genotype have a significantly higher rate of response to chemotherapy than those with the Ile/Ile or Ile/Val genotype [116]. A meta-analysis did not show any evidence of association of GSTP1 (Ile/Ile versus Val/Val) polymorphisms with prognosis in patients with OS [117]. OS Patients with poor response to chemotherapy, poor event-free survival, and poor overall survival have more GSTP1 rs1695 GG genotype and G allele [118]. Polymorphisms of other members of the GST family, such as GSTM1, GSTT1, GSTM3, were also described to play a role in treatment response and OS progression. GSTM1 with a null genotype have a poor clinical outcome compared with the GSTM1 genotype having at least one allele, which is associated with a good response to treatment and, therefore, a better survival rate. A better survival rate is observed when the patient has GSTT1 null genotype. Finally, the presence of metastases at diagnosis can be associated with the GSTM3*B allele [117,119]. Furthermore, GSTP1 may protect against cytotoxic effects of chemotherapy agents on OS cells. This can be mediated by GSTP1 overexpression (described above) but also by regulation of signaling pathways such as the mitogen-activated protein kinase (MAPK) or c-Jun N-terminal kinase (JNK) pathways. GSTP1 protects OS cells from CDP- or DOX-induced oxidative stress and, therefore, cell death by inhibiting JNK phosphorylation or by activating phosphorylation of extracellular signal-regulated kinase 1/2 (ERK1/2) and p38 MAPK [120,121,122]. Indeed, GSTP1 inhibition in OS cells reduced activation of ERK 1/2, leading to apoptosis and DNA damage [108]. Without oxidative stress conditions, JNK is sequestered in a protein–protein complex with the enzyme GST and, JNK cannot be phosphorylated, therefore, inhibiting apoptosis. In the case of oxidative stress following chemotherapy treatment, the JNK/GST complex is dissociated, inducing JNK phosphorylation and, therefore, apoptosis activation. In resistant cells, GST is overexpressed thus, JNK/GST complexes will be plentiful.

To overcome resistance due to GSTP1, 6-(7-Nitro-2,1,3-benzowadiazol-4-ylthio) hexanol (NBDHEX), a GSTP1 inhibitor was tested in vitro in OS cell lines that were resistant to CDP, DOX, and MTX. NBDHEX is effective in cells with higher GSTP1 levels and enzymatic activity [106]. However, NBDHEX is associated with cytostatic effects, but a positive effect on metastases development was observed [123]. A proteomic investigation showed that NBDHEX dissociated the GSTP1-tumor necrosis factor receptor-associated factor (TRAF) 2 complex, thus activating JNK and p38 and leading to DNA damage and apoptosis [124].

## 4. DNA Repair Improvement

Under physiological conditions, DNA can be damaged by endogenous or exogenous agents or by DNA polymerase, which can introduce errors during replication. Nevertheless, cells have mechanisms that allow them to reduce the deleterious effects of this damage. Damage tolerance, cell cycle checkpoints, DNA repair, and cell death are some of these mechanisms. Despite the presence of all these mechanisms, DNA damage may persist, leading to genomic instability of cells, which will then be eliminated via programmed cell death. After DNA damage, the cell makes cell cycle arrest in order to recruit proteins involved in the main DNA repair mechanisms such as direct reversal repair, base excision repair (BER), nucleotide excision repair (NER), mismatch repair (MMR), homologous recombination (HR), and non-homologous end-joining (NHEJ) [125].

Chemotherapeutic agents are known to cause DNA damage, leading to cell death. However, tumor cells can sometimes resist treatment by improving the DNA repair pathway. Resistance to CDP frequently leads to cross-resistance to other DNA damaging agents used in OS protocols, such as CDP, DOX, or IFO. That is why the most studied drug in the case of resistance due to increased DNA repair is CDP [96,126].

The resistance of OS to some chemotherapy agents may be due to an improvement in the BER mechanism, and in particular, by the over-regulation of one of the key enzymes, the apurinic endonuclease APE-1 [27]. Indeed, APE-1 has been associated with MDR and prognosis in many cancers [127]. It has been shown that over 70% of samples of OS patients have a high expression of APE-1. A correlation between the high expression of APE-1 and the decrease in survival rate has been shown [128]. A more recent study also showed overexpression of APE-1 in 65% of samples from OS patients, as well as an amplification of the APE1 gene. In addition, overexpression of APE1 has been linked to local OS recurrence and/or metastasis [129].

The efficacy of inhibitors of APE1, such as lucanthone, 7-nitroiondole-2-carboxylic acid, resveratrol, and arylstibonic acids, has been evaluated in vitro and to sensitize cells to treatment [130,131,132,133]. However, the efficacy of these inhibitors is quite low or nonspecific [127]. In OS cells, miR-513a-5p suppresses APE1 expression, making tumor cells radiosensitive [134]. MiR-765 downregulates APE1 and sensitizes OS cells and tumor xenografts to CDP [135]. The use of small interfering RNA (siRNA) targeting Ape1, pSilenceApe1, sensitizes OS cells and tumor xenografts to the antiangiogenic andostatin [136]. Thus, targeting APE1 with miRNA or siRNAs can be a treatment option for resistant OS.

Another important protein of the BER pathway, poly(ADP-ribose) polymerase 1 (PARP1), may be involved in OS chemotherapy resistance. In fact, high expression of PARP1 in OS is correlated with shorter survival [137]. The use of olaparib, a PARP1 inhibitor, or siRNA targeting PARP1 sensitizes OS cell lines to DOX. In addition, inhibition of proliferation and induction of apoptosis are more significant with co-treatment of olaparib and DOX [137]. A phase II clinical trial is in progress in order to evaluate the effect of olaparib alone in the treatment of refractory disease [138]. The effect of a combination of olaparib with ceralasertib, the ataxia telangiectasia, and rad3 related (ATR) kinase inhibitor is currently being studied in another phase II clinical trial including patients with unresponsive or recurrent OS [139].

The DNA repair pathway most often implicated in chemoresistance is NER [140]. Some members of the NER pathway have been studied, including DNA excision repair proteins, excision repair cross-complementing protein (ERCC). High levels of ERCC proteins are related to cisplatin resistance. Hattinguer et al. showed ERCC 1 positivity in 26% of OS patients, which was significantly associated with worse event-free survival and overall survival [141]. Low expression of ERCC4 was correlated with a poor histological response to chemotherapy [142]. Li et al. showed that the expression of ERCC4 and ERCC2 in OS cells was greater in patients with high tumor necrosis compared to patients with low tumor necrosis [143]. The study of polymorphisms of NER genes has shown the existence of a significant positive association between polymorphism ERCC2 gene, Lys751Gln but also ERCC1, Asn118Asn with the improved CDP response. In addition, these gene polymorphisms are associated with an increase in event-free survival rate [144,145]. ERCC1 rs11615CC alleles were linked with a better clinical outcome [146]. ERCC1 C8092A genotypes and event-free survival are positively correlated. Indeed, patients with the C allele (CC and CA) have significantly longer event-free survival rates than those carrying the AA genotype [147].

ERCCs can be targeted in order to increase the sensitivity of OS cells to chemotherapy agents. Sensitivity to CPD increases in the resistant U2-OS/CDDP300 and U2-OS/CDDP1 cells by silencing ERCC1, ERCC2, ERCC3, and ERCC4 genes [126]. NER gene inhibitors may also increase CDP sensitization in vivo. Indeed, NSC130813 and triptolide have improved the efficacy of CDP in resistant and sensitive cell lines [126] and deserve a clinical evaluation.

## 5. Cell Cycle and Apoptosis Disruptions

Chemotherapy causes DNA damage leading to cell death through apoptosis. Cell cycle stops allow cells to repair DNA damage. Cells, therefore, escape apoptosis and continue their cell cycle. Alterations of cell cycle signaling or apoptosis are partly responsible for chemotherapy resistance in tumor cells. In fact, modulation of chemotherapeutic cytotoxicity is due to cell cycle and apoptosis-related gene expression dysregulation. In resistant OS cells, apoptosis pathways are disturbed [148].

TP53 gene has an important role in cell cycle arrest as well as in apoptosis in case of DNA damage. This gene has been demonstrated to be involved in the modulation of anticancer drug cytotoxicity [149]. Deletions or mutations of TP53 promote the malignant characteristics of many cancers [150]. OS has a high level of P53 mutations [151]. However, the role of P53 in resistance to therapies in OS is controversial. Wild-type or mutant p53 genes were associated with the chemoresistance in OS cells [150]. P53-null Saos-2 cells were rendered resistant to DOX and MTX following transfection of a p53 mutant (TP53-R273H). In addition, these cells exhibited downregulation of apoptotic enzymes, such as pro-caspase 3, suggesting that resistance to P53-dependent apoptosis is the cause of loss of chemosensitivity [152]. When Murine Double Minute 2 (Mdm2) expression, a downstream mediator of p53, is enhanced, the p53-mediated apoptosis is inhibited, endowing cells with resistance to DNA-damaging agents [153]. Asada and his colleagues showed that a CDP-resistant OS cell line had a low level of P53 WT protein compared to the parental cell line [154]. Transfection of P53 WT into OS cells not expressing p53 increased their sensitivity to CDP [155].

The role of P53 in resistance to chemotherapy may vary depending on the extracellular conditions and soluble factors present. Under normal Fetal Bovine Serum (FBS) conditions (10%), P53 induction in Saos-2 cells resulted in a decrease in CDP sensitivity, while P53 induction under low FBS conditions (1%) resulted in an increase in CDP sensitivity [156]. In addition, over-expression of miR-140 is associated with chemosensitivity in OS xenografts, induced expression of P53, as well as cell cycle arrest in G1/G2 in OS cell lines with p53 WT. This observation is lower in cell lines with P53 mutated [157]. Contradictions are also present in clinical studies: One study, performed on 24 patient samples, showed loss of heterozygosity at the P53 locus in 54% of cases. In addition, in this group, only 15% of patients were sensitive to neoadjuvant chemotherapy compared to 64% of patients in the other group (where 46% do not present any changes at the P53 locus), suggesting that TP53 deletion was associated with decreased sensitivity and drug resistance [158]. Conversely, positive P53 expression was correlated with resistance or survival of OS patients. In fact, patients with low post-recurrence survival were those with P53 positive expression in lung metastasis samples [159,160]. A meta-analysis done on 499 patients showed that P53-positive patients tended to have poor 2-year survival rates. However, these results were not significant. P53 alteration was associated with a decreased survival, but P53 status was not correlated with the response to chemotherapy. Nevertheless, chemotherapy response was independent of the status of P53 [161]. No evidence that mutated p53 can predict the development of metastases, chemotherapy response, and clinical outcome in patients with high-grade OS was shown [162]. The p53-reactivating small-molecule RITA is able to sensitize certain tumor cells to chemotherapy, with a positive effect observed in patients with p53 mutations, while patients with a p53 deletion do not respond to this treatment [43]. The efficacy of RITA is currently being evaluated in OS as monotherapy or in combination with other therapy [43].

Cell death signaling is regulated by proteins belonging to the Bcl-2 family. This family of proteins contains anti-apoptotic proteins such as Bcl-2, Bcl-XL, and pro-apoptotic proteins, including Bax [163,164]. Bcl-2 and Bax affect drug-induced apoptosis and regulate chemotherapy resistance in various cancers. Chemosensitivity of OS cells to DOX and CDP could significantly be enhanced by inhibiting Bcl-2/Bcl-XL [165]. Conversely, pro-apoptotic protein’s up-regulation can thwart chemoresistance. Indeed, Bax up-regulation increases apoptosis and drug sensitivity of OS cells after etoposide treatment, while its downregulation reduces the chemosensitivity [166,167,168]. OS patients with high expression of Bcl-2 have a lower long-term survival rate compared to patients with low expression [169]. Patients presenting lung metastases had a higher frequency of Bcl-2 staining in primary tumor samples compared to patients without metastases. However, Bcl-2 high expression did not correlate with chemosensitivity or survival rate in OS patients [159,170,171]. Similarly, Bax expression does not predict overall or disease-free survival rate. However, patients with a high Bax/Bcl-2 ratio showed a decrease in 4-year disease-free and overall survival [171]. Targeting Bcl-2 family members sensitizes OS cells to chemotherapy agents. Indeed, a Bcl-xL inhibitor, navitoclax, inhibits proliferation in two canine OS cell lines [172] and deserves to be evaluated in human OS.

## 6. Involvement of Signaling and Signal Transduction Pathways

**Figure 2:**
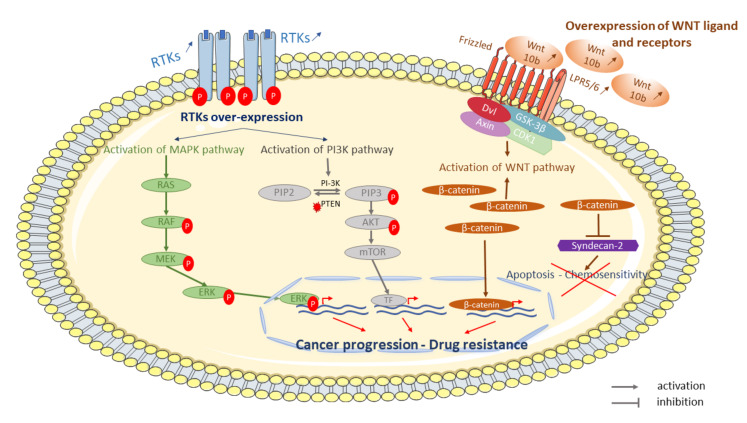
Some examples of signaling pathways involved in OS resistant tumor cells to conventional treatment. Cell surface-receptors, such as tyrosine kinase receptors (RTKs), are disturbed in resistant cells to chemotherapy. Over-expression and phosphorylation of RTKs activate signaling pathways such as mitogen-activated protein kinases (MAPK) or phosphoinositol 3-kinase (PI3K)-protein kinase signaling pathway. Activation of MAPK and/or PI3K leads to proliferation and survival, respectively. Several WNT ligands (WNT 10b) and receptors (lipoprotein receptor-related protein 5—LRP5) and β-catenin are over-expressed and activate the WNT/β-catenin pathway leading to β-catenin overexpression. β-catenin is able to repress syndecan-2 leading to inhibition of apoptosis and chemosensitivity.

Cell-surface receptors, like receptor tyrosine or serine/threonine kinase (RTK) have an important role in key biological processes such as differentiation, proliferation, and cell cycle control in normal and cancer cells [173]. These receptors are often disturbed and have a potential role in the emergence of chemotherapy resistance in OS. Indeed, it has been shown that resistant OS cells exhibit higher expression of several kinases compared with parental cell lines [126]. Over-expression of cell-surface receptors increases activation of signaling pathways such as mitogen-activated protein kinases (MAPK), which is highly activated in OS cell lines [174] (Figure 2).

### 6.1. Cell-Surface Receptors: Her2, VEGF and IGF-1R

The ErbB/HER family is one of the protein tyrosine kinases involved in chemotherapy resistance in OS. Indeed, immunohistochemistry and quantitative PCR analyzes showed overexpression of human epithelial growth factor receptor 2 (HER2) gene and Her2 protein in OS tumors [71,175], which is correlated with poor histological response and a poor survival [71]. High expression of VEGFR is also associated with worse disease-free and overall survival in OS [176,177]. A phase II clinical trial investigated the effect of trastuzumab, a HER2-targeted agent, in combination with cytotoxic chemotherapy in metastatic patients but did not show a difference between patients with HER2 overexpression and those without HER2 overexpression [178]. AZD2171, a VEGFR inhibitor, showed antitumor activity in OS xenografts and could, therefore, be in combination with conventional chemotherapeutic agents, a way of counteracting chemoresistance [179]. Insulin-like growth factor 1 receptor (IGF-1R) has been linked to OS pathogenesis and enhanced tumorigenesis in human and canine OS cell lines [180]. IGF-1R overexpression has been described in OS [181] and has been correlated with metastatic disease and poor overall survival [182], indicating that IGF-1R is a therapeutic target of interest in OS. Various studies have shown that IGF-1R inhibition using Tyrphostin (AG1024) combined with DOX or by targeting IGF-1R using shRNA enhances the effect of chemotherapy in OS cell lines [183,184]. A phase II clinical trial investigated the effect of a human neutralizing anti-IGF-1R antibody, Robatumumab, in patients with relapsed OS. None of the patients with unresectable metastases showed a response, and 3 of 31 patients with resectable metastases had a complete or partial response [185]. It would be interesting to know if the combination of this antibody with chemotherapy could be more effective.

### 6.2. PI3K and MAPK Pathways

Phosphoionositol 3-kinase (PI3K)-protein kinase B signaling pathway, which leads to cell survival and Ras, Raf, and ERK/mitogen-activated protein kinase (MAPK) pathway, which leads proliferation and tumor growth are activated by cell-surface receptors phosphorylation, such as IGF-1R [186]. These two pathways have also been demonstrated to be activated in OS cell lines [187]. Genome, exome, and RNA sequencing of 59 tumors showed mutations within the genes belonging to the PI3K/Akt/mTOR pathway, such as PTEN, TSC2, PI3KCA, PDPK1, AKT1, and EIF4B [188]. PI3K and MAPK pathways could be targeted in order to counteract the resistance of OS to chemotherapy agents. Akt inhibition with the allosteric Akt inhibitor MK-2206 was able to abrogate proliferation in U-2 OS and HOS cell lines [189]. The mTOR pathway presents molecular alterations in various cancers [190]. mTOR, a serine/threonine protein kinase, and its downstream target have been shown to be active in OS cell lines from dogs [191]. In human OS cell lines, rapamycin downregulated activity of mTOR and inhibited cell growth [192]. mTOR inhibition not only decreases survival but also plays a role in metastases development. Indeed, inhibition of the mTOR pathway using rapamycin or cell cycle inhibitor-779, a rapamycin analog, in the OS model significantly inhibits lung metastases development [193]. However, only dual inhibition of PI3K and mTOR induces apoptosis compared to the targeting of either PI3K or mTOR alone in OS cells [188]. A phase I clinical trial analyzing rapamycin effect on pediatric tumor panels, including OS, showed an anti-tumor activity [194], suggesting that the mTOR pathway can be targeted and could be combined with conventional chemotherapy to reduce or prevent the emergence of resistance. Targeting the MAPK pathway inhibits proliferation, invasion, metastasis development, and drug resistance in bone sarcomas [195]. PD-98059, an inhibitor of ERK1/2 phosphorylation, increases expression of proapoptotic proteins such as Bax and induces cell death in OS cell lines. ERK1/2 inhibition increases DOX sensitivity in OS cells, delays tumor growth, and prolongs survival by inducing pro-apoptotic proteins. The combination of DOX and PD-98059 prolongs the survival of mice with OS [195]. A clinical trial showed clinical benefits following MAPK/ERK inhibition combined with chemotherapy in patients with unresectable or metastatic OS [195].

### 6.3. WNT Pathway

The Wnt/β-catenin pathway plays a major role in many cellular processes such as proliferation, migration, polarization, and differentiation. The Wnt signaling pathway deregulation or mutations are involved in tumor development, resistance, and relapse. An increase in signaling activity Wnt/β-catenin pathway was observed in OS [196,197]. Wnt aberrant activation is implicated in the development and metastatic progression of OS [198,199,200] and is also involved in resistance development to therapies in OS [201]. Overexpression of several Wnt ligands, such as Wnt10b, and receptors, such as lipoprotein receptor-related protein 5 (LRP5) in OS tissues and cell lines, leads to increased pathway activation [188]. Furthermore, β-catenin, an intracellular wnt component, is highly expressing in OS tissues compared to healthy tissues. High expression of β-catenin is associated with poor prognosis and lung metastases formation [202]. DOX treatment of OS cells leads to syndecan-2, a proteoglycan capable of influencing apoptosis and chemosensitivity, overexpression. Syndecan-2 upregulation can be repressed by β-catenin activation reducing sensitivity to DOX in OS cells [203]. Aberrant signaling of the Wnt pathway could be due to a possible interaction between TRIM37 and β-catenin [204]. Samples of OS tumors showed over-expression of TRIM37 at mRNA and protein levels. TRIM37 overexpression in OS cells following treatment with CDP, DOX, MTX, or IFO induced drug resistance, while TRIM37 knockdown restored chemosensitivity. However, TRIM37-induced chemoresistance has been partially dependent on the activation of the Wnt/β-catenin signaling pathway [205]. Wnt/β-catenin pathway activation is also associated with the presence of cancer stem cells (CSCs) population, leading to recurrence of OS tumors, but also with cell transition to a cancer stem-like phenotype in OS cells following treatment with chemotherapy.

The wnt/β-catenin pathway could be used as a potential therapeutic target to increase drug sensitivity in OS. Small molecule inhibitor of Wnt/β-catenin, such as PRI-724, which suppresses Wnt/β-catenin-mediated transcription, was able to inhibit OS cell proliferation. Decreasing protein levels of the target Wnt, Cyclin D1, suppresses Wnt signaling [206]. Moreover, several Wnt-signaling drugs are being investigated in phase I clinical trials and showed promising outcomes [207,208].

## 7. Autophagy Involvement

Autophagy is a process of protecting and recycling cellular materials where cellular organelles and damaged proteins are degraded by autophagosomes. Autophagy thus makes it possible to maintain cellular biosynthesis. Autophagy allows cells to survive under cellular stress by removing organelles and proteins in order to provide energy [209]. Tumor cells often exploit the autophagy pathway in order to promote chemotherapy resistance and survival [210].

Indeed inhibition of autophagy using 3-methyladenine (3-MA) increased cell death induced by paclitaxel in OS cells [211], suggesting that a therapeutic combination of autophagy inhibitors with chemotherapy could be therapeutic alternatives in OS treatment. Zhang et al. have shown that downregulation of autophagy via 3-MA in MG-63 cells increased chemotherapeutic sensitivity of tumor cells treated with CDP [212]. Another study also showed that autophagy protects OS cells against cell death induced by therapies such as photodynamic therapy [213], meaning that autophagy can have a protective role in OS cells. High Mobility Group Box1 (HMGB1), a chromatin-binding nuclear protein, could facilitate autophagy after administration of cytotoxic agents in order to promote tumor cell survival [214]. HMGB1 mRNA overexpression has been detected in OS cells treated with DOX, CDP, and MTX. Furthermore, HMGB1 overexpression increases autophagy and chemoresistance in vitro [215]. Indeed, HMGB1 knock-down restores sensitivity to chemotherapy of OS cells and increases apoptosis. HMGB1 is also overexpressed in tumor xenografts allowing tumor cells to resist apoptosis in the presence of chemotherapies [216]. Moreover, HMGB5 overexpression leads to over-regulation of autophagy and to chemotherapy resistance, while HMGB5 knock-down downregulates autophagy and sensitizes cells to chemotherapy agents [217]. HSP90AA1 expression was also found to increase drug resistance in OS by inducing autophagy and inhibiting apoptosis. Expression of HSP90AA1 is up-regulated after treatment with chemotherapy agents, such as CPD, DOX, and MTX. Indeed, suppression of HSP90AA1 restored sensitivity to chemotherapy both in vivo and in vitro [218]. Autophagy is a resistance mechanism that can be targeted in order to develop new therapies. Chloroquine could be a new therapeutic strategy in OS treatment by increasing apoptosis and promoting tumor cell death in various cancer, such as lymphoma or colon cancer, through autophagy inhibition [219,220]. Chloroquine blocks the autophagic process in cisplatin-resistant OS cells [221]. Rapamycin combined with chloroquine increase the antitumor efficacy in OS cells [222].

## 8. Cancer Stem Cells and Microenvironment

The efficacy of anticancer therapy, such as chemotherapy, can be influenced by the tumor microenvironment [223]. Indeed, the presence of CSCs but also an acidic tumor microenvironment can cause MDR in the tumor.

CSCs or tumor-initiating cells are a small population of tumor cells, which could drive cancer initiation, propagation, metastasis, and relapse of cancers. These cells share common properties with normal stem cells (SCs). CSCs can self-renew while producing differentiated daughter cells, and their quiescent nature has been shown to be involved in mechanisms of MDR [224]. CSCs are found in a specific microenvironment comparable to a niche of traditional stem cells. This niche may affect the ability of CSCs to grow, renew themselves, invade, metastasize, and hypoxic stability, providing additional protection against anticancer therapy for CSCs [225]. Different hypotheses have been expressed regarding the role of CSCs in the therapeutic resistance of OS. Indeed, populations of stem-like cells are present in OS cell lines and in OS biopsies. In addition, these cells expressed mesenchymal stem cell markers such as CD133, CD117, CD105, and CD44 [226,227,228]. These subpopulations are resistant to chemotherapy agents. A study showed that CSCs cell line, 3AB-OS, selected from human OS MG-63 cells by long-term treatment with 3-aminobenzamide (3AB), were positive for a pluripotent stem cell marker, CD133 and exhibited higher expression of the ATP-binding cassette subfamily G member 2 (ABCG2) transporter gene involved in drug efflux, as well as higher expression of anti-apoptotic genes, including Bcl-2, leading to resistance [229]. An increased expression of drug efflux transporters P-gp and BCRP (Breast Cancer Resistance Protein)/ABCG2 in a population of OS CSCs derived from the HOS-MNNG cell line has been shown, suggesting that stem cells play a role in chemotherapy resistance of tumors [230]. Some OS cell lines, such as MG-63, are capable of forming sarcosphers. These sarcospheres resemble CSCs due to their ability to self-renew and expression of stem cell-related genes and DNA repair enzymes [231]. CSCs and sarcospheres showed a strong chemoresistance to DOX and CDP due to increased expression of DNA repair enzyme genes. Resistance could be overcome by administering a DNA repair enzyme inhibitor, caffeine [231]. MG-63 sarcospheres also exhibited increased mRNA levels of ALDH1, a protein involved in enhancing drug detoxification, which is correlated to drug-resistant [232]. Inhibition of some resistance mechanisms described in this review, such as inhibition of drug efflux systems, alteration of enhanced DNA repair pathways, inhibition of detoxification, promoting factors pro-apoptotic, and the inhibition of anti-apoptotic factors, could be considered to target OS SCCs.

OS cells overexpress S-Phase kinase-associated protein2 (skp2), known to positively regulate CSC populations and self-renewal ability [233]. In MTX-resistant OS cells, overexpression of skp2 was found to promote epithelial–mesenchymal transition (EMT) and enhance invasive, migratory, and attachment abilities. Skp2 inhibition using shRNA increased the sensitivity of tumor cells to MTX [234]. Compound 25, an inhibitor of Skp2, has antitumor activities and cooperates with conventional chemotherapy to suppress cancer cell survival [233]. Nitidine chloride represses the expression of EMT markers and, therefore, inhibits proliferative, migratory, and invasive abilities of OS cell lines (U2-OS) [235]. These two compounds may have a potential interest in the treatment of OS.

The acidity of the OS microenvironment could be partly responsible for drug resistance. Sensitivity to DOX can be reduced by lowering extracellular pH in p-gp negative OS. Indeed, DOX combined with a proton pump inhibitor, omeprazole, reduces tumor volume in the OS xenograft model. Reversing the pH gradient contributes to the sensitization of cells to DOX but also to CDP and MTX [235]. This means that the reduction in acidity in the tumor microenvironment could, in part, overcome resistance in OS.

Different drugs have been tested as monotherapy to treat OS. Trabectedin, a chemotherapeutic agent that binds to DNA and causes damage and apoptosis, showed no effect when used as monotherapy, while the use of Trabectedin combined with conventional chemotherapy agents could be beneficial and suppress resistance-associated genes [236]. Indeed, Trabectedin reduces tumor growth and metastases development in the OS model [237]. In addition, Trabectedin promotes binding of Runx-2 transcription factor leading to osteogenic differentiation. The mOS69 mouse model (the most aggressive model) does not normally exhibit extensive T cell infiltration. However, Trabectedin allows the recruitment of CD8+ and CD4+ T cells and, combined with anti-PD-1 antibody, significantly inhibits tumor growth in the mOS69 model [237]. This finding could be adapted and evaluated as new treatment options in patients with refractory or recurrent OS.

## 9. Conclusions

A better knowledge of resistance mechanisms responsible for treatment failure in some patients allows identifying new strategies or therapeutic targets in order to adapt therapies to patients. Personalized medicine, including targeted therapies as well as immunotherapy, offers new possibilities to counteract resistance to conventional treatments for patients with cancer. In addition, an innovative approach, the Antibody-Drug Conjugate (ADC) strategy, is also under investigation. This technology consists of grafting a cytotoxic agent onto a monoclonal antibody, which is able to bind an antigen on the surface of cancer cells. After internalization of ADC, the cytotoxic drug damages the cancer cell. These ADCs have the advantage of targeting and killing tumor cells while sparing healthy cells, thus limiting side effects and improving patients’ quality of life. ABBV-085, an antibody-drug conjugate targeting LRRC15, has shown promising results in sarcomas. Glembatumumab vedotin (also known as CDX-011) targeting GPNMB (GlycoProtein Non-metastatic Melanoma protein B) was also evaluated in a pivotal phase II clinical trial (NCT02487979) in OS and was shown to have an effective antitumor activity. New personalized therapeutic approaches are now being studied and deserve to be evaluated to limit or avoid the emergence of resistance and improve the therapeutic management of patients with OS.

## Data Availability

The study did not report any data.
